# UKHCDO gene therapy taskforce: Guidance for implementation of haemophilia gene therapy into routine clinical practice for adults

**DOI:** 10.1111/hae.15125

**Published:** 2024-11-20

**Authors:** Pratima Chowdary, Beatriz Duran, Paul Batty, Gillian Lowe, April Jones, Debra Pollard, Sara Boyce, Jayashree Motwani, Bahareh Amirloo, Kathryn Musgrave, David Hopper, Stephen Classey, Sarah Whitaker, Nicola Dunn, Annette Bowyer, Susan Shapiro

**Affiliations:** ^1^ Katharine Dormandy Haemophilia and Thrombosis Centre Royal Free Hospital London UK; ^2^ Department of Haematology Cancer Institue University College London London UK; ^3^ Pharmacy Department Manchester University NHS Foundation Trust Manchester UK; ^4^ West Midlands Adult Comprehensive Care Haemophilia Centre Queen Elizabeth Hospital Birmingham UK; ^5^ Haemophilia Centre Royal Victoria Infirmary The Newcastle upon Tyne Hospitals NHS Foundation Trust Newcastle upon Tyne UK; ^6^ University Hospital Southampton Haemophilia Comprehensive Care Centre Southampton UK; ^7^ Department of Haematology Birmingham Children's Hospital Birmingham UK; ^8^ Centre for Haemostasis and Thrombosis Guy's and St Thomas NHS Foundation Trust London UK; ^9^ Southern Haemophilia Network Basingstoke and North Hampshire Hospital Basingstoke UK; ^10^ Department of Coagulation Royal Hallamshire Hospital Sheffield UK; ^11^ Oxford Haemophilia and Thrombosis Centre Oxford University Hospitals Oxford UK; ^12^ Radcliffe Department of Medicine Oxford University Oxford UK

**Keywords:** clinical practice guidelines, gene therapy, haemophilia A, haemophilia B, multidisciplinary, patient pathway, UKHCDO

## Abstract

**Introduction:**

2022 was a landmark year with two adeno‐associated viral vectors (AAVs) receiving conditional marketing authorization from EMA for the treatment of persons with severe haemophilia A and severe to moderately severe haemophilia B and a third in 2024. Gene therapy is a transformative, irreversible treatment with long‐lasting effects, necessitating development of new clinical pathways to ensure optimal outcomes.

**Aim:**

To develop a consensus framework and service specification for delivery of AAV gene therapy for haemophilia in adults within the UK using the hub‐and‐spoke model proposed by the European Association of Haemophilia and Allied Disorders and the European Haemophilia Consortium.

**Methods:**

The UK Haemophilia Centre Doctors Organisation (UKHCDO) set up a working party to develop expert consensus guidance, working with NHS England to ensure alignment with NHS England commissioning and the national service specification.

**Results:**

These guidelines detail the patient pathway, counselling and governance requirements for the hub‐and‐spoke model. The national service specification requires the hub site to manage governance for AAV‐based gene therapy. Proposed regional and national multidisciplinary teams will harmonize clinical practices incorporating expertise from various specialities and professional groups. Key requirements identified include standardized documentation and multidisciplinary collaboration. Nationally agreed patient information and counselling checklists will streamline the informed consent process and facilitate data collection for long‐term safety and efficacy monitoring.

**Conclusion:**

These guidelines provide a structured framework for the delivery of liver‐directed gene therapy. Whilst specific to the United Kingdom they provide a framework for the implementation of gene therapy in other countries for haemophilia and other monogenic disorders.

## 1 INTRODUCTION

Patients with severe haemophilia A (HA) and haemophilia B (HB), the most common severe bleeding disorders with X‐linked inheritance, now can expect a near‐normal life expectancy due to therapeutic advances and comprehensive care. The last decade has seen a phenomenal increase in the available therapeutic options, addressing many unmet needs, enhancing bleed protection and improving quality of life.^1^ Haemophilia is a paradigm for the management of monogenetic disorders, and current treatment options can be categorized into three groups: factor replacement therapies, non‐replacement therapies and gene therapy.^2^ Currently, there are no established criteria that help determine the optimal initial therapeutic option or when to switch treatments. In this context, clinicians and patients face two key decisions: the first is between replacement and non‐replacement therapies, and the second is between reversible and irreversible treatment. Discussion of the advantages and disadvantages of various treatment options, alongside patient preference, is crucial to identifying the right treatment for the right patient.^3^

Gene therapy involves the introduction of a functioning gene, editing a mutated gene to treat disease, or inactivating a gene to decrease harm.^4^ Gene therapy is unique for its long‐term effects and the irreversible nature of the intervention. The aim is to achieve extended, possibly lifelong relief from disease symptoms. Haemophilia exemplifies in‐vivo gene therapy, where the therapeutic gene (transgene) is delivered directly to the patient using a liver‐directed viral vector.^5^, ^6^ Effective treatment requires safe and effective transgene delivery to the liver, ensuring protein expression at levels sufficient to ameliorate the disease phenotype.

Recombinant adeno‐associated viral (rAAV) vectors are the most commonly used vectors in haemophilia treatment. Wild‐type AAVs (wt‐AAV), on which these are based, are small, single‐stranded DNA parvoviruses, around 25 nm in diameter and 4.7 kb in size.^7^ Wt‐AAV is unable to replicate and requires co‐infection with a helper virus for its propagation. AAV vectors are popular due to their non‐pathogenic nature, that is, infections are mostly asymptomatic and the presence of multiple serotypes with distinct tissue tropisms.^8^, ^9^ AAV can be engineered to carry a transgene in the place of its original viral sequences, creating recombinant AAV (rAAV) vectors. Furthermore, the innate immune response to AAV is mild and short‐lived with minimal clinical impact, making them attractive vectors for gene therapy.^10^

### 1.1 Haemophilia gene therapies

Recombinant AAV gene therapy (GT) vectors for the treatment of haemophilia use a single infusion of a liver‐targeting rAAV vector containing a functional copy of either the factor VIII or IX gene, which results in their localization to hepatocytes and subsequent expression. The first successful gene therapy trial using liver‐directed rAAV demonstrated the effectiveness of peripheral vein infusion.^11^, ^12^ Since then, multiple recombinant vectors have been trialled with a few notable failures.^13^, ^14^, ^15^

In 2022, the European Medicines Agency granted conditional marketing authorization for the first gene therapy treatments in adult males (18 years or more) for severe HA, valoctocogene roxaparvovec, Roctavian®^16^ and HB etranacogene dezaparvovec, Hemgenix®,^17^ with a third one fidanacogene elaparvovec, Durveqtix®^18^ granted conditional marketing authorization in 2024. Additionally, there are several gene therapies at earlier stages of development.

The therapies differ in the AAV capsid, transgene modifications, manufacturing methods and type of immunosuppression used. There is a notable variation in dosing across different studies, although a dose‐response relationship is evident within individual trials. Several factors appear to influence factor levels, including vector dose, vector manufacturing platform, host immune response, non‐immune cellular responses and other less understood factors.^13^ This remains an area of active research. Some key differences between the licensed products are described in Table 1, and readers are directed to the other reviews for details.^19^, ^20^, ^21^

**TABLE 1:** Haemophilia gene therapies currently licensed.^a^

	Valoctocogene roxaparvovec	Etranacogene dezaparvovec	Fidanacogene elaparvovec
Brand name	Roctavian	Hemgenix	Beqvez (Durveqtix)
Indication	Severe haemophilia A	Severe & moderately severe haemophilia B	Severe & moderately severe haemophilia B
Dose (vg/kg)	6e13	2e13	5e11
Vector capsid	AAV5	AAV5	AAV‐Rh74var (SPK100)
Transgene	ss‐CO‐FVIII‐BDD‐SQ	ss‐CO‐FIX‐R338L (Padua)	ss‐CO‐FIX‐R338L (Padua)
Production cell line	Sf9	Sf9	HEK293
AAV antibody	Exclusion criteria	Caution if ≥1:678.	Exclusion criteria
Phase 3 Study ClinicalTrials.gov	GENEr8‐1 NCT03370913	HOPE‐B NCT03569891	BENEGENE‐2 NCT03861273
Approvals	EMA (conditional) FDA	EMA (conditional) FDA Health Canada	EMA (conditional) FDA Health Canada

Abbreviations: AAV, adeno‐associated virus; CO, codon‐optimized; EMA, European Medicines Agency; FDA, US Food and Drug Administration; ss, single‐stranded.

^a^ Approvals are limited to some geographical regions.

## 2 GENE THERAPY IN HAEMOPHILIA—IMPORTANT CONSIDERATIONS

Certain aspects of gene therapy that are pertinent to short‐ and long‐term outcomes are discussed below.^7^, ^19^, ^22^

### 2.1 Seroprevalence of anti‐AAV antibodies and eligibility

Seroprevalence refers to the proportion of adults and children with antibodies against AAV in their plasma, indicating previous exposure or infection. UK seroprevalence studies in severe HA demonstrated anti‐AAV5 and anti‐AAV8 antibodies in 30% and 40% of patients, respectively.^23^ These antibodies include neutralizing antibodies that cross‐react with rAAV vectors and prevent the transduction of cells and gene transfer,^24^ and non‐neutralizing antibodies. Early clinical trials showed that pre‐existing immunity to AAV was associated with absent or impaired response,^9^ with trials excluding patients with detectable antibodies. However, the Phase 3 trial of etranacogene dezaparvovec enrolled patients with anti‐AAV antibodies, with low titres not affecting gene transfer but high titres linked to treatment failure.^17^

Anti‐AAV antibodies can be detected and quantified by enzyme linked immunosorbent (ELISA) and transduction inhibition assays.^25^ Assays for these antibodies are expected to be provided by the gene therapy manufacturer through a central lab or a designated specialized laboratory. It is important to note that the lack of standardization of anti‐AAV antibody assays impedes meaningful comparisons across gene therapy studies. Standardized approaches associating assay results with clinical outcomes are still needed.

### 2.2 Vector‐induced immune response and transaminitis

In haemophilia gene therapy trials, the vector‐induced immune response often manifests as an asymptomatic increase in liver transaminases, referred to as transaminitis, with alanine aminotransferase (ALT) levels rising earlier and more significantly than aspartate aminotransferase (AST) levels. The immune response encompasses both innate and adaptive immunity that can target the viral vector, the transgene and its product.^26^ Transaminitis, coupled with a loss of factor levels, has been linked to an increase in capsid‐specific T cells approximately eight weeks after vector infusion.^9^, ^27^ The frequency of transaminitis varies between studies, and the immune response to rAAV appears to be influenced by the vector dose, type and transgene.^13^, ^14^ Importantly, immunosuppression has been used to manage transaminitis and stabilize factor levels.^9^, ^27^

Corticosteroids are the most commonly used immunosuppressive medications for managing and preventing immune responses. They have widespread inhibitory effects on both innate and adaptive immune cells. They can be initiated prophylactically, typically 2–4 weeks after gene therapy infusion, or reactively in response to transaminitis.^12^, ^28^ Reactive approaches require vigilant follow‐up for prompt identification and treatment, while prophylactic strategies can reduce the intensity of follow‐up but increase the risk of adverse effects from immunosuppression.

Nevertheless, there is no clinical data to support one strategy over another; for licensed products, the approach should be based on the summary of product characteristics. Once initial transaminitis is controlled, there have been no reported instances of late recurrences leading to loss of transgene expression. Prediction, prevention and management of this immune response merits further research.^12^, ^28^, ^29^

### 2.3 Factor response: Variability in magnitude and durability

Haemophilia gene therapy trials have demonstrated a dose‐response relationship, with higher vector doses leading to increased gene expression. Marked interindividual variability has been observed, and potential mechanisms have been extensively reviewed in other publications.^26^ Additionally, the vector doses used across different trials are not directly comparable due to differences in vector type and manufacturing processes.

Specifically for haemophilia, there are notable differences in the duration of response for FVIII compared to FIX gene therapy.^19^ FVIII expression appears to decrease over time, even in the absence of transaminitis, unlike the more stable expression observed with FIX. The reason for this slow and late decline in FVIII expression is unclear, but potential explanations include the loss of the relatively large FVIII transgene from transduced hepatocytes (FIX transgene is much smaller) or silencing of the FVIII transgene.^30^, ^31^

### 2.4 Lab monitoring

FVIII:C and FIX:C levels measured post‐gene therapy show variability across different reagents. FVIII activity post valoctocogene roxaparvovec shows a 1.6‐fold higher result with the one‐stage APTT‐based clotting FVIII assay (OSA) compared to the chromogenic substrate assay (CSA).^32^, ^33^ There is no conclusive evidence for which assay best reflects the in vivo FVIII activity, but most gene therapy programmes now exclusively report FVIII:C using CSA.

Similarly, FIX levels after expression of the high activity Padua FIX variant post etranacogene dezaparvovec and fidanacogene elaparvovec treatment show up to 2.5‐fold higher levels with OSA than CSA. However, the FIX CSA has limited laboratory availability and limited clinical applicability and should not be used for the routine monitoring of FIX transgene expression.^34^ Moreover, variability between APTT reagents in the OSA has been reported in laboratory field studies of the Padua FIX molecule.^34^, ^35^

Therefore, careful selection of assays and reagents for  FVIII or FIX monitoring post gene therapy is necessary to reduce the factors that can contribute to variability. Wherever practicable, the same activity assays used in the trials should be used to monitor response to treatment, which is particularly pertinent when patients are monitored at more than one centre.

### 2.5 Long term safety

The primary long‐term safety concern for all gene therapy vectors, including AAV, is whether these have the potential to affect liver health or contribute to the risk of cancer development. Following infection with wild type AAV, the viral genome persists in a latent state in both non‐integrated (episomal) and integrated forms within host cells.^36^, ^37^, ^38^ No significant long‐term sequelae of wt‐AAV have been identified, which is supported by a high prevalence of seropositivity. Recently, a possible minor role of wt‐AAV infection in the development of hepatocellular carcinoma has been hypothesized.^39^, ^40^ Differences in the structures of therapeutic AAV vectors compared to wt‐AAV limit the translation of these studies to current gene therapy vectors, which do not contain viral sequences.^41^ Studies of rAAV in animal models have shown that vectors predominantly persist in episomal forms with low levels of integration. Although studies performed in neonatal mice described increased incidence of hepatocellular carcinoma with a specific integration site (*Rian* locus), this has not been seen in other animal models or in biopsies obtained from clinical studies.^42^, ^43^, ^44^ In the context of haemophilia gene therapy, six cases of cancer have been reported to date, with detailed molecular studies not demonstrating any causality.^45^, ^46^ At present, although these risks remain theoretical, there is a need for registries and life‐long follow‐up of patients post‐gene therapy.

## 3 CARE DELIVERY: HUB AND SPOKE MODEL

Any care model should ensure equitable access to gene therapy across the nation. The European Haemophilia Consortium (EHC) and the European Association for Haemophilia and Allied Disorders (EAHAD) have published guidelines outlining the key principles of care.^47^, ^48^ A hub and spoke model has been proposed as a practical approach due to the limited number of centres with clinical trial experience and the expected low annual treatment numbers. This model aims to build and share clinical expertise for the best patient outcomes. The hub and spoke model allows patients to receive care at local haemophilia centres while benefiting from the expertise of an experienced hub. While this model promotes cost‐effectiveness and equity of access, it may limit the development of widespread clinical expertise and require some patients to travel significant distances, incurring extra costs and time.

In the United Kingdom, certain Haemophilia Comprehensive Care Centres (CCCs) have been designated as gene therapy dosing sites or hubs. These hubs and spoke flows are usually not fixed, though established networks and ‘natural flows’ from specific sites to a Gene Therapy Hub typically exist. The delineation of responsibilities between hubs and spokes is based on each centre's experience and is expected to evolve. Their roles may be locally modified based on experience.

### 3.1 Hub centres

Hub centres typically have experience in licensed gene therapy and/or clinical trials. They are responsible for the procurement, storage, prescription and administration of gene therapy medicinal products (GTMP). Additionally, they provide staff and facilities for liaising with spokes, establishing local pathways, supporting patient consent, and facilitate administration and monitoring of gene therapy. Hub centres play an active role in the consent process, including reconsenting pre‐infusion and obtaining consent for the gene therapy registry. Moreover, the hub treatment centres must have the infrastructure and facilities to ensure the safe administration of the gene therapy product, including patient monitoring and management of any infusion reactions. They are expected to run regional multidisciplinary team (MDT) meetings, attend national panel meetings and oversee data reporting for the long‐term follow‐up registry.

### 3.2 Hub multidisciplinary (MDT) team

**FIGURE 1:**
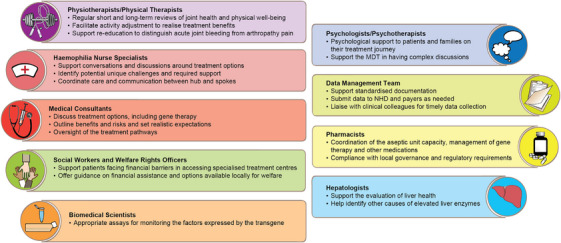
Multidisciplinary team at the hub.

The comprehensive management of gene therapy for haemophilia requires a multidisciplinary team approach for patient‐centred care. A prerequisite for the establishment of the MDT team is the education of various healthcare professionals, as discussions with patients require a degree of specialist knowledge and the use of decision coaching and aids. A full MDT team needs to be established at the Hub, and the various MDT members and their roles are described in Figure 1.

### 3.3 Regional MDT meetings

Regional Multidisciplinary Teams (MDTs), consisting of the hub and connected spokes, must be multi‐professional and include a variety of specialists, including designated hepatologists. The hub or regional MDT will be responsible for ongoing patient care. These MDT meetings will serve as forums to review and ratify local agreements, evaluate capacity within the hub‐spoke framework, establish communication strategies, and develop joint policies on roles, responsibilities and escalation procedures. They must establish robust processes that comply with data protection for data sharing, discussion and timely escalation of abnormal results. Additionally, regional MDTs can meet more frequently to support the coordination of patients moving through the pathway locally.

Regional MDTs will receive completed patient proformas from spoke centres, including results for anti‐AAV antibodies. The MDT will assess eligibility and potential benefits for individual patients. Upon confirming eligibility, information will be forwarded to the national panel, and a patient‐specific treatment plan will be initiated. This plan will include baseline investigations and assessments, a psychological review and a discussion of practical logistics.

## 4 MANAGEMENT AND REGULATION OF ATMPS

Advanced Therapy Medicinal Products (ATMPs), as defined by the European Medicines Agency, are biological medicines for human use that include gene therapy, somatic cellular therapy, tissue‐engineered products and combinations with medical devices.^49^ The majority of the currently licensed gene therapies are classified as Genetically Modified Organisms (GMOs) class I‐II. Gene therapy medical products (GTMPs) are composed of a vector or delivery formulation/system containing a genetic construct that is engineered to express a specific transgene for the regulation, repair, replacement, addition or deletion of a genetic sequence.^50^

In the United Kingdom, the Chief Pharmacist at dosing centres holds the responsibility for the governance and management of ATMPs, akin to other medicinal products. National guidelines detail the role of pharmacy in delivering ATMPs, including their roles and responsibilities and the procedures for handling and preparing both ex‐vivo and in‐vivo GTMPs.^51^, ^52^ The Pan UK Pharmacy Working Group for ATMPs recommends the conduct of a risk assessment and evaluation of any GTMP by the Genetic Modification Safety Committee (GMSC) or equivalent as the preferred organizational governance route for licensed products.

**FIGURE 2:**
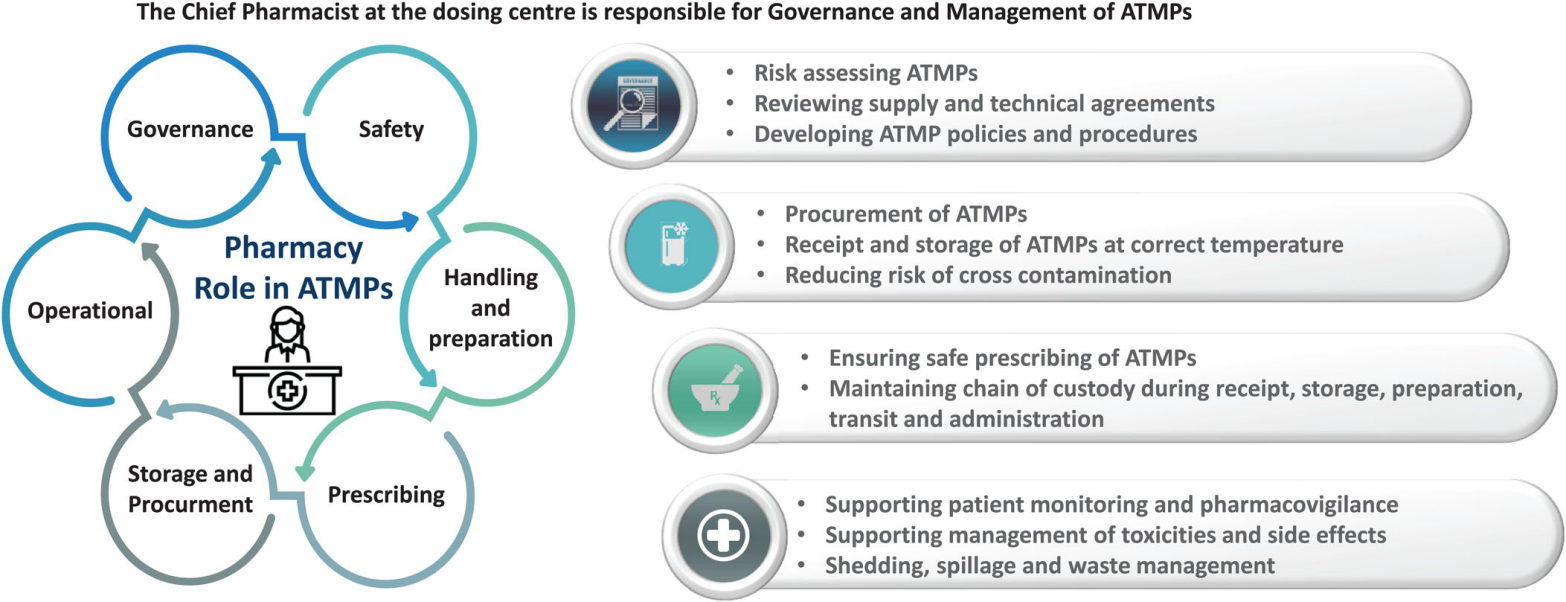
ATMP governance and pharmacy oversight.

Each dosing centre should establish an organizational governance process clearly outlined in its local ATMP policy. Additionally, a well‐defined standard operating procedure (SOP) for the management of ATMPs should be in place. These centres must possess the necessary facilities for receiving, storing, preparing, administering, and disposing of GTMPs and any related waste, ensuring the gene therapy's quality is suitable for its intended use (Figure 2).

Ideally, in‐vivo GTMPs should be handled and prepared in the pharmacy to mitigate any risk related to preparation and medication errors. Preparation risk assessments should be conducted when new ATMPs are introduced to aid in determining the most appropriate preparation location.^53^ The preparation location may vary based on the reconstitution requirements before administration, the stability of the reconstituted product, and the training levels and competency of staff members involved. Consideration can be given to handling the product in the clinical setting provided appropriate risk assessments have been undertaken that include relevant information from the Summary of Product Characteristics and biological containment level required followed by the development of local SOPs.^51^, ^52^

## 5 PATIENT INFORMATION, SHARED DECISION MAKING AND CONSENT

Discussions about gene therapy should include all the issues detailed in Table 2.^54^ During these discussions, communication challenges should be considered, and sources of information should be provided that are appropriate to literacy/comprehension level and health literacy.^55^ Particular attention should be given to the following issues:

1. **Benefits of treatment**: The primary benefit is the potential for long‐term relief from bleeding tendencies, including cessation of routine prophylaxis, reduction of treatment burden and improvement in quality of life.
2. **Variability in response and clinical impact**: The discussion should highlight the challenge of predicting post‐treatment steady‐state factor levels for individual patients. A change in clinical phenotype to moderate or mild haemophilia offers a more practical approach to treatment decisions than factor levels. Generally, those classified as mild may not require prophylaxis, while those in the moderate category will need individualized assessment, with most not requiring prophylaxis.
3. **Treatment failure and potential for retreatment**: It is important to highlight the potential for treatment failure, characterized by a lack of response or loss of response over time. Currently, gene therapy is considered a once‐in‐a‐lifetime treatment due to the development of neutralizing antibodies post‐treatment, which may hinder the effectiveness of future gene therapies. While research on retreatment options is ongoing, no immediate solutions are available.
4. **Durability of expression**: Patients should be aware of the inter‐individual variability in the duration of expression, particularly the differences between haemophilia A and haemophilia B, with the latter showing stable long‐term expression.
5. **Long‐term safety**: While data up to ten years show no association between adeno‐associated virus (AAV) therapy and cancer, ongoing registries and studies are crucial for continuous safety monitoring. It is important to emphasize the unknown long‐term risk, especially concerning hepatocellular cancer.
6. **Patient expectations and motivation**: It is crucial to manage patient expectations by emphasizing the unpredictability of individual outcomes. An ideal result would be living treatment‐free and potentially symptom‐free, with significant long‐term relief from the burden of treatment. However, it is also important to address the potential for disappointment in cases of treatment failure or non‐response. Additionally, the discussion should cover how treatment may impact relationships with friends and family.
7. **Time commitment and potential lifestyle restrictions**: Emphasize the intensity of the initial follow‐up, which includes restrictions on long‐distance travel and potential impact on work and personal life. Discuss the side effects of steroids on well‐being, mood and weight gain. Mention the necessity to avoid alcohol for the first 6–12 months and its potential impact on work and social life. Discuss the need for barrier contraception even if the female partner of reproductive age is on the combined pill because of vector shedding. Although dual barrier contraception is more effective it is not mandated for the current licensed products.
8. **Psychological support**: Highlight the feedback from patients in clinical trials regarding the need for such support.
9. **Other current and future therapeutic options**: Shared decision‐making should present gene therapy as one component of a comprehensive therapeutic strategy. Evaluate the risks, benefits and effectiveness of alternative therapies, including the option to delay treatment while awaiting future gene therapy advancements.

**TABLE 2:** Counselling checklist for consent for gene therapy.

Information to be covered during discussions
Gene therapy as a treatment option Mechanism of action and irreversible nature of the intervention Unique features compared to other treatment options
Potential benefits—general Average response per gene therapy product Impact on haemophilia Cessation of regular factor prophylaxis if and when endogenous factor levels are around 5 IU/dL or greater On‐demand treatment for bleeds and surgery, determined by factor levels achieved
Response to treatment—what can be expected Factor levels that can be achieved and inter‐individual variability Duration of response and inter‐individual variability Differences in the duration of response between HA and HB Potential for lack of response (non‐responder) and loss of response with time (treatment failure) Immune response to vector and inability to re‐treat with the same gene therapy vector
Potential outcomes post–gene therapy Typically evaluated at 6–12 months when stable expression is likely to have been achieved In the event of no response, reiterate the need to continue with the current treatment Conversion to moderate haemophilia; factor levels between 1 and 5 IU/dL, with scope for cessation of prophylaxis or reduced frequency of prophylaxis with on‐demand treatment, particularly if levels are 3 IU/dL or greater Conversion to mild haemophilia; factor levels greater than 5 IU/dL, when prophylaxis will be stopped, and treatment with clotting factors used on‐demand for bleeds and surgery Good to excellent response; factor levels > 15–20 IU/dL, where treatment is likely to be required for major surgery only
Risk of thrombosis if levels in the supraphysiological range Patients with severe haemophilia appear to have reduced mortality from cardiovascular disease and venous thromboembolism compared to the general population. Increasing factor levels are likely to increase an individual's risk to the population mean, particularly in those with additional risk factors, e.g., smoking, hypertension, high cholesterol, and diabetes Interventions will require an individualized ongoing risk assessment
Psychological aspects The rationale for choosing gene therapy and patients' life circumstances, particularly the need for time commitment, behaviour changes and regular reviews Patient's expectations about what success and failure look like
Immediate side effects of infusion Allergic reactions Headache, nausea Flu‐like illness and fatigue
The intensity of follow‐up: initial and long‐term Frequency of investigations during the first 6 months, particularly the need for weekly investigations for the first 3–6 months Additional visits if concerns about liver enzyme elevation Yearly liver health monitoring with liver ultrasound scan and blood tests as required Yearly follow‐up for a minimum of 15 years and potentially for the lifetime of the patient
Temporary lifestyle changes Avoidance of alcohol, recreational drugs and herbal supplements Potential challenges of long absences during the period of intense monitoring Impact on work Contraception, pregnancy and lactation advise should be given as per product‐specific SmPC and personal individual circumstances of the patient including the need for barrier contraception
Vector shedding (spread of vector to other body tissues, including semen) Male patients should be informed of the need for contraceptive measures for them or their female partners of childbearing potential as per the details provided in the license Patients treated with gene therapy must not donate blood, organs, tissues and cells for transplantation
Immune response, liver inflammation, and immunosuppression Impact of immune response on factor levels and need for close monitoring, particularly in the first 3–6 months Immunosuppression Rationale Reactive/prophylactic as per summary of product characteristics Medications used, mainly steroids Duration of treatment Side effects of immunosuppression, e.g., for steroids—weight gain, indigestion, problems sleeping, feeling restless, changes in mood Review current medications and switch to less hepatotoxic medications if appropriate and feasible Causes of elevated liver enzymes, including exercise, alcohol and immune response The rationale for avoiding potentially hepatotoxic agents for the first year (including alcohol, potentially hepatotoxic herbal products and nutritional supplements)
Long term safety Baseline screening for liver health and review by hepatologists as needed Unknown risk of liver cancer—theoretical basis and the lack of documented cases to date Advise on the need for long‐term liver monitoring with an annual liver ultrasound Participation in a long‐term registry for both safety and efficacy
Consent for registries Importance of long‐term registries and consent for the exchange of information with national and international databases

## 6 PSYCHOLOGICAL SUPPORT FOR PATIENTS CONSIDERING OR UNDERGOING GENE THERAPY

The lack of adequate psychological support has been highlighted as a significant issue by clinical trial participants in post‐treatment qualitative studies. Some individuals report feeling ‘haemophilia‐free’, while others experience a ‘loss of identity’.^55^, ^56^ Psychological support should begin pre‐infusion with discussions focused on patients' understanding of the physical and psychological demands of the treatment, managing expectations around outcomes post‐treatment and identifying any dissonance between patient hopes and potential clinical outcomes.

A psychological review also provides insights into patients' values, strengths and difficulties to enable tailored support. Psychological support helps patients manage family relationships and important conversations with partners and family members.^57^ Support should also be provided to those who are either clinically ineligible or declined for other reasons.^58^ It is critical that these evaluations and insights are shared with the wider MDT to support patient care.

Post‐treatment, psychologists, along with the MDT, can help patients manage the experience, constraints, outcome uncertainty, and any drug side effects, including those related to immunosuppression.^56^, ^59^, ^60^ They can also assist patients in coping with the impact on their personal identity, haemophilia management, lifestyle and relationships.^57^ Support is also necessary when patients come to terms with the initial outcome and potential long‐term uncertainty,^61^ as well as for managing distress and disappointment if their outcomes do not meet their expectations.

## 7 PATIENT PATHWAYS

**FIGURE 3:**
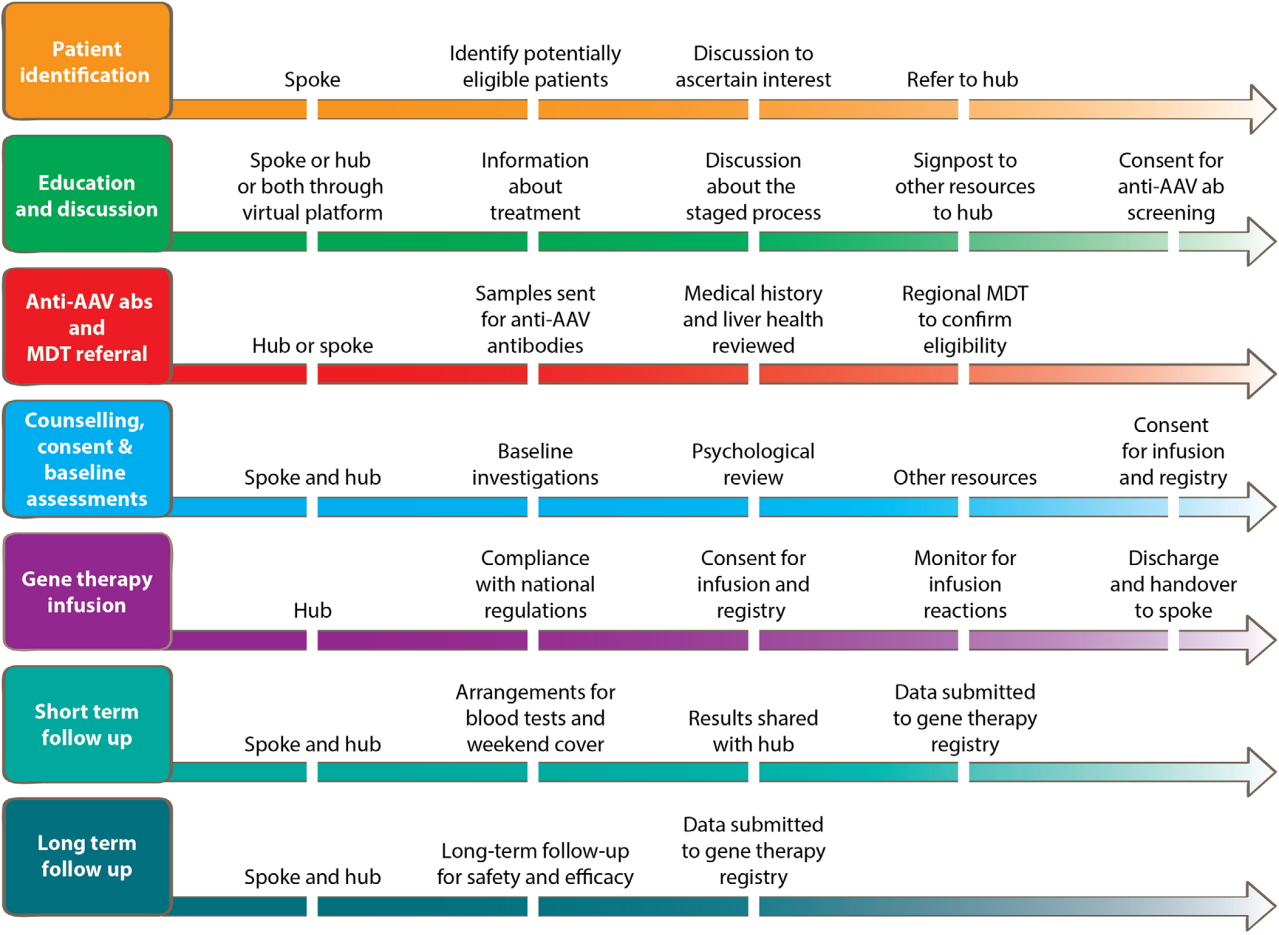
Patient pathway across the hub and spoke.

Successful gene therapy administration necessitates several critical steps: identification of patients likely to benefit from the treatment, provision of education and counselling, confirmation of eligibility and practical considerations, including treatment administration and follow‐up. Moreover, meticulous coordination between dosing hubs and haemophilia treatment centres is essential to delineate responsibilities clearly. Figure 3 illustrates the key steps in the patient pathway during gene therapy treatment and the potential role of hub and spokes.

### 7.1 Patient identification, education and discussion

Information about gene therapy is widely available in the haemophilia community. Patient discussions about gene therapy and eligibility may start long before formal decision‐making starts.^62^ Once licensed and commissioned, the option for gene therapy should be discussed with all potentially eligible patients by the centres (Table 3). This might be done through the pre‐screening of notes or in the clinic, as there are limited exclusion criteria. Patients must be provided with appropriate information, and centres must ensure that this information is provided in a way accessible to the patient and their families.

**TABLE 3:** Eligibility and assessments.^a^

Current inclusion criteria Severe HA (FVIII < 1 IU/dL) Severe and moderately severe HB (FIX ≤ 2 IU/dL) Potential for clinical and patient benefit No history of current or previous inhibitors that required immune tolerance
Current exclusion criteria Severe hepatic impairment (acute or uncontrolled chronic hepatitis, advanced fibrosis or cirrhosis) Active infection (acute or chronic) Immunosuppression (within the last 30 days) Hypersensitivity to the product excipients Age less than 18 years old Women with childbearing potential Limited life expectancy due to conditions such as active cancer and moderate‐severe heart failure due to the initial treatment burden and long‐term benefit Current inhibitor
Special populations Presence of anti‐AAV antibodies is an exclusion for some, but not all, gene therapies HIV is not a contraindication, although patients would need careful counselling and review of the antiretroviral therapy for drug interactions, as only a limited number of patients with controlled HIV have been included in the clinical trials^66^
Baseline history Haemophilia history Current treatment Current bleed control Inhibitor history Factor VIII or IX mutation Joint procedures to date Medical history Allergies Previous allergy to blood products Medical comorbidities
Baseline assessments Height and weight Psychological review MSK assessment Joint score (HJHS) Quality of life measures Haemophilia Activities List (HAL) EQ5D MSK assessment optional 6‐min walk test Timed up and go (TUG) Point of care USS (HEAD‐US) if available
Baseline investigations Baseline bloods Full blood count Renal function Liver enzymes (ALT, AST) Virology (hepatitis B/C/HIV) Anti‐AAV antibody results^b^ Factor level and inhibitor screen Baseline liver ultrasound and fibroscan (within the last 6 months)^b^

^a^ This is likely to be updated as new information becomes available,

^b^ within 6 months of the infusion date.

### 7.2 AAV antibody screening and MDT referral

The AAV antibody status should be assessed after the initial discussion if an eligible patient shows interest. A proportion of patients may be ineligible for treatment due to anti‐AAV antibodies and early testing reduces disappointments and delays. For individual products, there may be a timescale for test validity before repeat testing is required.

Counselling and education can be conducted by a spoke centre or hub, depending on their experience, or jointly by both teams. The counselling should cover the items listed in the counselling checklist provided in Table 2. If AAV antibody levels confirm eligibility, the spoke centre should conduct a formal eligibility review (Table 3). Patients should be encouraged to discuss the information with their family and friends. Managing patients' expectations and including a psychologist can make the consent process robust.

Individuals who are eligible for gene therapy and wish to proceed should be formally referred to the regional MDT for confirmation, and details should be provided to the national panel. A proforma should support this referral to ensure all relevant details are captured.

### 7.3 Counselling, consent and baseline investigations

Once eligibility has been confirmed, a follow‐up discussion six weeks or later after the initial discussion should confirm the patient's understanding and expectations before written consent. This can be through face‐to‐face or virtual appointments and should review all aspects covered previously, with a focus on (1) why gene therapy, (2) why now and (3) the safety considerations, with explicit documentation of the benefits, risks and potential for treatment failure.

In addition to the discussions, baseline assessments of musculoskeletal health, quality of life and liver health, as detailed in Table 3, need to be performed. Of note, baseline liver function tests should be done at least twice at any laboratory which will be used during the first year of follow‐up in order to inform the patient's baseline level and variability. This is also an opportune moment for a psychological review, if not initiated already. Patients should be advised to maintain a lifestyle that supports liver health, which includes avoiding alcohol, recreational drugs and over‐the‐counter herbal supplements for at least 6 months or as recommended in the individual product information. This precaution is to prevent incidental elevations due to alcohol excess being misconstrued as an immune response, resulting in repeat testing and potentially inappropriate immunosuppression.

### 7.4 Gene therapy infusion

Following patient consent, logistics should be discussed between the hub, spoke and patient regarding the infusion date, need for overnight accommodation, support provided by the hub and dates and locations for post‐infusion monitoring. This depends on the patient's home location relative to the hub and spoke sites and available local facilities. Where patients travel significant distances, suitable accommodation should be provided for the patient and one companion to stay overnight before/after the dosing. Support from social workers or welfare rights officers may be needed if additional funding is required.

The medical team must assess the patient's fitness for infusion, review eligibility and document written informed consent for the administration of gene therapy and potentially inclusion into the registry before GTMP preparation. An ultrasound within 6 months is acceptable, but it is prudent to ensure no abnormalities in liver function tests 2 weeks before infusion, with samples taken pre‐infusion.

The dosing site can order the gene therapy product following local procedures and policies following consent and confirmation of a date. The product's name, dose and batch number should be recorded for traceability. The gene therapy product must be administered in a setting with staff and equipment, including a spillage kit and resuscitation trolley, available to treat infusion‐related adverse reactions. If the GTMP is classified as a GMO, waste should be handled according to local procedures for GMO waste disposal, as outlined in the GTMP risk assessment, to ensure traceability as per local policy.

Infusions are given at the recommended rates, and patients are monitored closely for reactions. If an infusion reaction is suspected, the infusion should be slowed or paused. Antihistamine or corticosteroid treatment may be considered based on clinical judgment. The patient should be monitored for at least 3 h post‐infusion. If unplanned overnight monitoring is required due to the timing of gene therapy or an adverse reaction, an inpatient overnight stay on a suitable ward must be arranged.

Before discharge, the patient should receive a card or letter describing the treatment, along with contact details for both the hub and spoke in case of delayed reactions, and details for the initial follow‐up blood tests. A clear plan for factor prophylaxis should be given: recombinant factor prophylaxis will usually continue until endogenous factor levels increase (approximately >5 IU/dL). Patients should also be given a prescription for oral prednisolone to keep at home, with actual use determined by the regimen specified in the accompanying product literature.

### 7.5 Post infusion follow‐up

The following monitoring is proposed, which may vary from the individual product literature but helps establish a routine. The SmPC should be consulted for the final adjustments.

1. **Month 1–3**: Twice weekly investigations: FBC, renal, liver, AST, CK, FVIII/FIX levels using a suitable reagent. As there are discrepancies between one stage and chromogenic assays for FVIII and FIX gene therapies, it is generally advised to check individual product literature for the most suitable assay.
2. **Months 3–6**: Weekly investigations are dependent on individual product literature or if there are any liver function concerns; otherwise, monthly investigations are as above.
3. **Months 6–24**: Transition to monthly investigations (as above) for the first 6 months and quarterly thereafter. Liver ultrasound at 12 and 24 months.
4. **Years 2–15**: Six monthly evaluations of liver function tests. Annual liver ultrasound.

Consideration must be given to the impact of inter‐lab variability on liver function and factor‐level results. Ideally, the same laboratory must be used for liver functions at baseline and monitoring over time (particularly the first 3–6 months) to minimize the impact of inter‐laboratory variability. Some patients may likely need baseline liver function tests at both spoke and hub to allow flexibility with follow‐up. Blood results should be shared, and abnormal results should be escalated according to locally established protocol.

### 7.6 Elevated transaminases and immunosuppression

An elevation in liver enzymes should trigger consideration of repeating blood tests (and AST, CK, LDH) within 24 to 48 h and checking for alcohol consumption, hepatotoxic medications and exercise. The ALT cut‐off to introduce steroids was variable across studies and a course of steroids should be strongly considered in the event of an increase in ALT to greater than twice the baseline level or 1.5× the upper limit of normal. Some studies have used a lower value of 1.5× the baseline value and individual product literature should be consulted. Early discussion at the Hub MDT, National Panel and discussions with hepatology are helpful. Steroids administered are oral prednisolone 1 mg/kg or a fixed dose of 60 mg daily. There is experience of more rapid control with intravenous methylprednisolone, but the long term impact is unclear.^28^ The role of alternative immunosuppressants, including steroid‐sparing agents, to minimize side effects is an area of active interest with no concrete recommendations.^63^, ^64^

## 8 NATIONAL HAEMOPHILIA GENE THERAPY EXPERT PANEL

To ensure optimal patient care and ongoing learning, a National Haemophilia Gene Therapy Expert Panel will be established, which has core membership from each of the Gene Therapy Hubs in the United Kingdom. The panel will provide a forum for discussing the eligibility of complex patients and conducting an ongoing review of the patient's eligibility. Clinicians from the spoke site and other experts may be invited to join or participate in the panel as deemed necessary and relevant. As well as core members representing each hub site, membership will include core healthcare professional groups in medical, nursing, physiotherapy, pharmacy and psychology. The national panel offers opportunities for discussion with other groups, including hepatologists.

The panel is expected to meet regularly to identify issues that might benefit from shared and broader input and will work in partnership with regional Hub MDTs. It will also be a forum to discuss any challenges anticipated by the hub or spoke, collate adverse events for reporting to MHRA, discuss their management in conjunction with the adverse event working party, and provide oversight of the data collection. The National Panel will also identify significant issues requiring escalation to commissioners or clinical teams.

## 9 NATIONAL HAEMOPHILIA DATABASE AND GENE THERAPY REGISTRY

In the United Kingdom, the National Haemophilia Database (NHD) has an essential role in the pharmacovigilance of new therapies. Long‐term follow‐up in post‐marketing studies and registries is crucial to support the long‐term monitoring of AAV vectors to identify any potential safety issues that were not observed in preclinical or pivotal studies. The World Federation of Haemophilia (WFH) has established an international registry with the aim of determining the long‐term safety and efficacy of FVIII and FIX gene therapy.^65^ The NHD data fields, at a minimum, will include items recommended by the WFH gene therapy registry and other items of local interest. Consideration needs to be given to establishing long‐term follow‐up clinics within hubs linked to the national MDT and NHD.

As with other established treatments, the NHD will be the primary source of data relating to long‐term safety and efficacy. Hubs and Spokes will need to work closely with each other to ensure that patient contact is maintained in the long term for ongoing management of the patient's haemophilia. Although the principal responsibility for data collection post‐gene therapy resides with the Hub, input and support from the patient's local care provider (the spoke) is required.

## 10 CONCLUSION

Gene therapy in haemophilia is now a reality in routine clinical practice. The potentially irreversible nature of the intervention justifies the development of a new clinical framework for consent and treatment administration. The hub and spoke model emphasizes the importance of patient‐centred collaboration. Ongoing collaboration between centres and shared data management is essential for addressing the challenges of long‐term monitoring of patient safety and outcomes.

## AUTHOR CONTRIBUTIONS

The UKHCDO Gene Therapy Working Group members were multi‐professional and included Haematologists—Pratima Chowdary (chair), Sara Boyce, Jayashree Motwani, Paul Batty, Kathryn Musgrave, Gillian Lowe and Susan Shapiro; Pharmacists—Beatriz Duran and Amiraloo Bahareh; Haemophilia nurse specialists—April Jones and Debra Pollard; Haemophilia physiotherapists—David Hopper and Stephen Classey; Haemophilia psychologists—Sarah Whitaker, Nicola Dunn and Haemophilia laboratory scientist—Annette Bowyer. The first draft included sections provided by various subgroups. Pratima Chowdary drafted the final version. All authors reviewed the final drafts and final version and approved it for submission. Declaration of generative AI and AI‐assisted technologies in the writing process: During the preparation of this work, the author(s) used ChatGPT version 4.0 to harmonize English writing style. The authors have reviewed and edited the final version and take full responsibility for the content of the publication.

## CONFLICT OF INTEREST STATEMENT

Pratima Chowdary has received research funding from Bayer, CSL Behring, Freeline, Novo Nordisk, Pfizer, SOBI; honoraria from BioMarin, CSL Behring, Chugai, Novo Nordisk, Pfizer, Roche, Sanofi, Sobi and Takeda; contributed to advisory boards for Bayer, Boehringer Ingelheim, Apcintex, CSL Behring, Chugai, Freeline, Novo Nordisk, Pfizer, Roche, Sanofi, Spark, Sobi, Takeda; received travel support from CSL Behring, Novo Nordisk, Pfizer, Sobi and Takeda; Paul Batty has received research support from Biomarin, honoraria from Biomarin, Octapharma, Pfizer, CSL Behring and Novo Nordisk; and travel support for conference attendance from CSL Behring and Octapharma. Jayashree Motwani has received an educational grant and speaker fees from Roche, Sobi, Bayer and CSL Behring. Sara Boyce has received speaker fees from CSL Behring, AstraZeneca and Sanofi; research support from Sangamo Therapeutics LTD; and sponsorship from CSL Behring for advisory meeting. Annette Bowyer has received speaker and advisory honoraria from Pfizer. April Jones has received speaker fees from Novo Nordisk, LFB and Sobi, has been on the advisory board for Pfizer, and has received sponsorship to attend conferences from Roche/Chugai. Debra Pollard has acted as a paid consultant to BioMarin relating to the production of education materials and has received honoraria from Roche‐Chugai and Sobi for speaking at educational meetings. Gillian Lowe has received research fellow funding from Biomarin and honoraria for participation in medical education events from CSL Behring, Sobi, Alexion, Abbvie, Leo, Novo Nordisk, Takeda, Amgen, Novartis and Sanofi. Susan Shapiro has received conference support from CSL Behring, Roche and Sobi; speaker fees from Chugai, Sobi and Takeda; consultancy fees from CSL Behring, E‐therapeutics and Sobi; and research grant support from Bristol Myers Squibb. Susan Shapiro receives funding support from the Medical Research Council (MR/T024054/1). Stephen Classey received a speaker fee from Roche, Sobi and a grant from Pfizer. Nicola Dunn has acted as a consultant to Pfizer. Bahareh Amirloo, Beatriz Duran, David Hopper and Sarah Whitaker have no competing interests.

## STAKEHOLDERS

The final draft was circulated to the UKHCDO advisory committee, NHS England, Industry partners and haemophilia society for comments.

## ETHICS STATEMENT

No human participants were included in this review and ethical approval was not required.

## Data Availability

Data sharing is not applicable to this article as no datasets were generated or analysed during the current study.
